# Sources of individual differences in adults’ ICT skills: A large-scale empirical test of a new guiding framework

**DOI:** 10.1371/journal.pone.0249574

**Published:** 2021-04-19

**Authors:** Alexandra Wicht, Stephen Reder, Clemens M. Lechner

**Affiliations:** 1 University of Siegen, Siegen, Germany; 2 Portland State University, Portland, OR, United States of America; 3 GESIS–Leibniz Institute for the Social Sciences, Mannheim, Germany; Universitat de Valencia, SPAIN

## Abstract

We develop an integrative conceptual framework that seeks to explain individual differences in the ability to use information and communication technologies (ICT skills). Building on practice engagement theory, this framework views the continued usage of digital technologies at work and in everyday life (ICT use) as the key prerequisite for the acquisition of ICT skills. At the same time, the framework highlights that ICT use is itself contingent upon individual and contextual preconditions. We apply this framework to data from two recent German large-scale studies (*N* = 2,495 and *N* = 2,786, respectively) that offer objective measures of adults’ ICT skills. Findings support our framework’s view of ICT use as a key prerequisite for ICT skills. Moreover, they demonstrate that literacy skills have strong associations with ICT skills, largely by virtue of their indirect associations through ICT use. By comparison, regional digital cultures (as proxied by internet domain registration rates) evince only limited explanatory power for individual differences in ICT skills.

## ICT skills in the information age

Across the past two decades, ICT skills—that is, the ability to use information and communication technologies—have gained currency for individuals and societies alike [1]. Recent studies, for example, attest to an important role of ICT skills for individuals’ employability, earnings, and social participation—as well as societies’ economic growth [e.g., 2–4]. As a consequence of this digital transformation, ICT skills have become a new fault line along which social inequalities emerge.

Given the growing importance of ICT skills, the question arises as to the factors that predict individual differences in ICT skills. However, extant evidence on adults’ ICT skills is sparse, scattered, and lacks theoretical coherence. In this study, we develop a unified conceptual framework that consolidates previous theorizing and research and aims to gain a better understanding of the correlates of individual differences in adult ICT skills. Toward that end, our framework seeks to identify the most important correlates (i.e., potential determinants) of ICT skills. Building on practice engagement theory [5] and related theoretical ideas, our framework assumes that the continued usage of ICT in the contexts of work and everyday life (henceforth “ICT use”) is a key prerequisite acquiring and maintaining ICT skills, thus highlighting the role of mostly informal learning processes. At the same time, building on the findings of previous studies the framework directs attention to the fact that ICT use is itself contingent upon a range of individual and contextual preconditions, most notably individuals’ literacy skills (i.e., reading competence) and the socio-cultural practices related to ICT in the wider regional context.

We put this framework to a test in two large-scale, high-quality studies on adult skills in Germany. Both studies measured the key constructs stipulated by our framework with comparable—albeit not identical—measurement instruments. Moreover, both studies addressed essentially the same target population, despite some differences in the age distribution because of the sampling frame and the waves under study. Analyzing both datasets in parallel allowed for a built-in replication of our findings. If both datasets lead to the same findings despite the differences in the specific measures used and the sample composition, this would greatly bolster our confidence in these findings.

## Previous evidence on the sources of individual differences in adults’ ICT skills

Although there is an ongoing debate on the so-called “digital divide” or “digital divides”—the “haves” and the “have-nots”—regarding access to and usage of digital technologies in the information age and knowledge society [6–8], little is known about the factors that drive adults’ actual ICT skills. Only a few studies draw on objective, standardized measures of adults’ ICT skills to identify these skills’ potential determinants [9–12]. Other studies investigate individuals’ subjectively assessed ICT skills [13–17]. The latter set of studies must contend with the problem of self-report bias since previous research shows that subjectively and objectively assessed ICT skills correlate only moderately [18–20]. Besides being sparse, evidence on adults’ ICT skills is also highly scattered. Existing evidence comes from different disciplines and has focused on widely varying sets of potential determinants of ICT skills.

Research on the sources of individual differences in adults’ ICT skills has identified two groups of factors that are thought to be possible determinants of an individual’s ICT skills: socio-demographic characteristics and ICT use. The first type of study emphasizes the role of education, age, sex, and migration. Typically, lower-educated, older, female and immigrated individuals are found to have lower ICT skills on average [8, 11, 13, 21–24]. The precise mechanisms behind the associations of these socio-demographic characteristics with ICT skills, however, are not entirely clear. The second group of studies looked beyond socio-demographic characteristics to also address the role of behavioural factors, highlighting the role of individuals’ ICT use at work and/or in everyday life, usage that positively correlates with digital skill [9, 10, 12, 14, 25–31]. These are the first studies that focus on how access, usage and ICT skills are related to each other. They suggest that ICT use is a key prerequisite for acquiring and maintaining ICT skills, championing the role of informal learning practices.

At the same time, it is important to note that ICT use is itself contingent on several preconditions [see 32, 33 for exceptions]. In this regard, previous studies on ICT use have shown that besides traditional socio-demographic correlates of ICT use—age, gender, education, and ethnicity [34, 35]—the regional macro-context may play an important role in shaping individuals’ ICT use. This is evidenced, for example, by a major urban-rural gap in the access to ICT [36–39]. Moreover, Brynner et al. [26] point to the importance of micro-contexts, such as home and workplace, in structuring individuals’ ICT use. Thus, both ICT skills and ICT use should be seen as dependent on individual socio-demographic characteristics and the multi-layered contexts in which individuals’ lives unfold. Together, socio-demographic characteristics and life contexts determine individuals’ access to ICT, their patterns of ICT use, and hence their opportunities for acquiring and maintaining ICT skills. From this perspective, ICT use can be regarded as a key mediator of the relationship between socio-structural opportunities and individuals’ ICT skills.

In the following, we offer theoretical perspectives that allow us to integrate the correlates of ICT skills identified by previous research—socio-demographic characteristics, ICT use, and socio-structural or contextual opportunities—into a unified conceptual framework. This framework gives centre stage to ICT use as a precondition for acquiring ICT skills while highlighting that ICT use is itself contingent upon a range of individual and contextual factors.

## Theoretical perspectives on adults’ ICT skills

### ICT use as a prerequisite to the acquisition of ICT skills

Practice engagement theory [5, 40], which was developed in the context of literacy research (see Reder, Gauly and Lechner [41] for a recent application to numeracy), aims to explain how individual differences in proficiency arise by highlighting the role of practice in everyday settings such as work or home. It states that skills such as literacy and numeracy in general, and ICT skills in particular, reciprocally interact with individuals’ engagement in information-processing practices, such as using the computer or the internet for information acquisition, documentation or presentation of contents or communication with others [26]. Practice engagement states that such practices allow individuals to expand their skills, which in turn tend to further reinforce attendant practices, instigating a self-reinforcing cycle of practice and skill acquisition.

Practice engagement theory is in line with constructivist learning theories [42, 43] according to which learning is rooted in the learner’s activities. Constructivist learning theories champion the role of non-formal and informal learning processes, that is, learning processes outside formal settings that do not lead to certificates. Such informal learning processes are thought to gain particular importance during adulthood after individuals have finished their formal education [44, 45].

The social practices view espoused by practice engagement theory and by constructivist learning theories is particularly apt for explaining differences in ICT skills. This is because current cohorts of adults, especially the older ones, typically received no or only little formal training in ICT skills during their formative years. Instead, for them, the acquisition of ICT skills depended heavily on “learning-by-doing”—that is, on the extent to which they used ICT at home and/or at work [34, 46].

### Contextual opportunities for ICT use and digital skill acquisition

If recurring practice engagement–that is, ICT use–is central to the acquisition of ICT skills, the question then arises what are the sources of ICT use itself? Non-formal and informal learning processes are strongly contingent upon the learning opportunities offered by an individual’s social context. Contextual opportunities play an important role in the social cognitive theory [47, 48] as well as in practice engagement theory [49, 50], both of which highlight the relevance of context-specific role models and social practices in shaping individuals’ learning opportunities.

Which social contexts, then, offer relevant opportunities for adults to use ICT and, consequently, to acquire ICT skills? As noted earlier, learning during adulthood mainly takes place outside of formal educational settings. Regarding ICT skills, most learning takes place in a multitude of non-formal and informal learning environments. These learning environments can be regarded as distinct yet interrelated micro-contexts [51]. The workplace is arguably the most central context in which adults make use of ICT to fulfill given tasks. For instance, adults can learn to deal with complexity, generate, formulate and evaluate options, access knowledge, seek expert help with or without the aid of ICT [52]. Following practice engagement theory, the continued use of ICT to manage given work tasks will, over time, result in higher ICT skills.

Furthermore, Bronfenbrenner’s ecological systems theory [51] directs attention to the fact that micro-contexts such as work or the family do not operate in a vacuum but are embedded in a set of more distal contexts that shape, and are in turn shaped by, different micro-contexts. In this regard, the regional context becomes relevant, as individuals’ behaviour in general, and their ICT use in particular, are always locally situated [53]. Regional contexts provide the “digital infrastructure” for using ICT in terms of access to high-speed internet [36, 54], but, what might be more important, regional contexts are characterized by different “digital cultures” [55]. These digital cultures are objectified in regional-specific social practices that may encourage or discourage individuals and organizations from integrating digital technologies into their everyday practices by setting relevant social norms and providing role models [56, 57]. Following social cognitive theory [47, 48] and adoption and diffusion theories influenced by it [58, 59], social learning is the most important driver of individuals’ behaviour, including attitudes and beliefs [8], and, in general, social change. That is, individuals in environments in which a stronger “digital culture” encourages them to adopt digital technologies may be more likely to adopt and use these technologies themselves, which in turn will positively influence the development of their ICT skills over time.

### Literacy as a prerequisite to ICT use and digital skill acquisition

Focusing on the individual level, there seems to be a consensus among scholars that individuals’ ICT use, as well as their ICT skills, is strongly dependent on cognitive abilities [1, 60]. In this regard, literacy skills can be seen as the most crucial set of cognitive skills, besides mathematical and problem-solving competences or the ability to think critically. Literacy skills refer to the ability to decode and comprehend written language. Following the literacy hypothesis ([61]; for an in-depth discussion see [62]), literacy skills can be assumed to be an indispensable prerequisite to using ICT and hence to acquiring ICT skills. After all, digital technologies are heavily based on text and abstract symbols that need to be processed and decoded. Consider, for example, a simple web search through Google or any other search engine. While this might seem a basic task for readers of this journal, it can be virtually inaccessible for individuals who cannot read and understand written information. In this regard, van Deursen and van Dijk [33] found that literacy skills particularly impact on formal internet use (e.g. navigating the internet by using hyperlinks), information internet use (e.g. locating required information, selecting and evaluating information) and strategic internet use (e.g. taking advantage of the internet by developing an orientation towards a particular goal or taking the right action to reach this goal). Furthermore, the study by Desjardins and Ederer [9] found that reading and writing (i.e., literary practices) on the job and in everyday life were positively related to individuals’ objectively measured ICT skills. Hence, literacy skills need to be considered as a precondition for ICT use and, by virtue of this, for the acquisition of ICT skills.

Despite the obvious centrality of literacy skills for ICT skills, literacy skills have rarely been taken into account in research on the determinants of ICT use and ICT skills [13, 33]. Even so, in the debate on (functional) literacy of the last decade, literacy skills and ICT skills are more and more seen as interrelated entities [63, 64]. Coiro [65] points out that the notion of literacy as individuals’ ability to read, write and understand the meaning of the content of conventional print media only, is no longer sufficient, as the internet provides new text formats and new ways to gain information that challenge individuals who only learned to read paper-based media [66].

## Toward an integrative guiding framework

### Outline of the framework

In sum, major theories of skill acquisition such as practice engagement theory assign a key role to social practices—more specifically, ICT use—in settings such as the workplace or everyday life. As we argued, ICT use is likely to be of particular relevance to the acquisition of ICT skills because current cohorts of adults typically received little or no formal training in ICT skills and acquired them through non-formal and informal learning processes, that is, through “learning by doing.” However, ICT use itself depends critically on several prerequisites. Chief among them are the opportunities and encouragements to engage with ICT offered by the multi-layered contexts in which individuals live on the one hand, and individuals’ endowment with literacy skills, which are indispensable in order to engage with (heavily text-based) digital technologies on the other.

**Fig 1:**
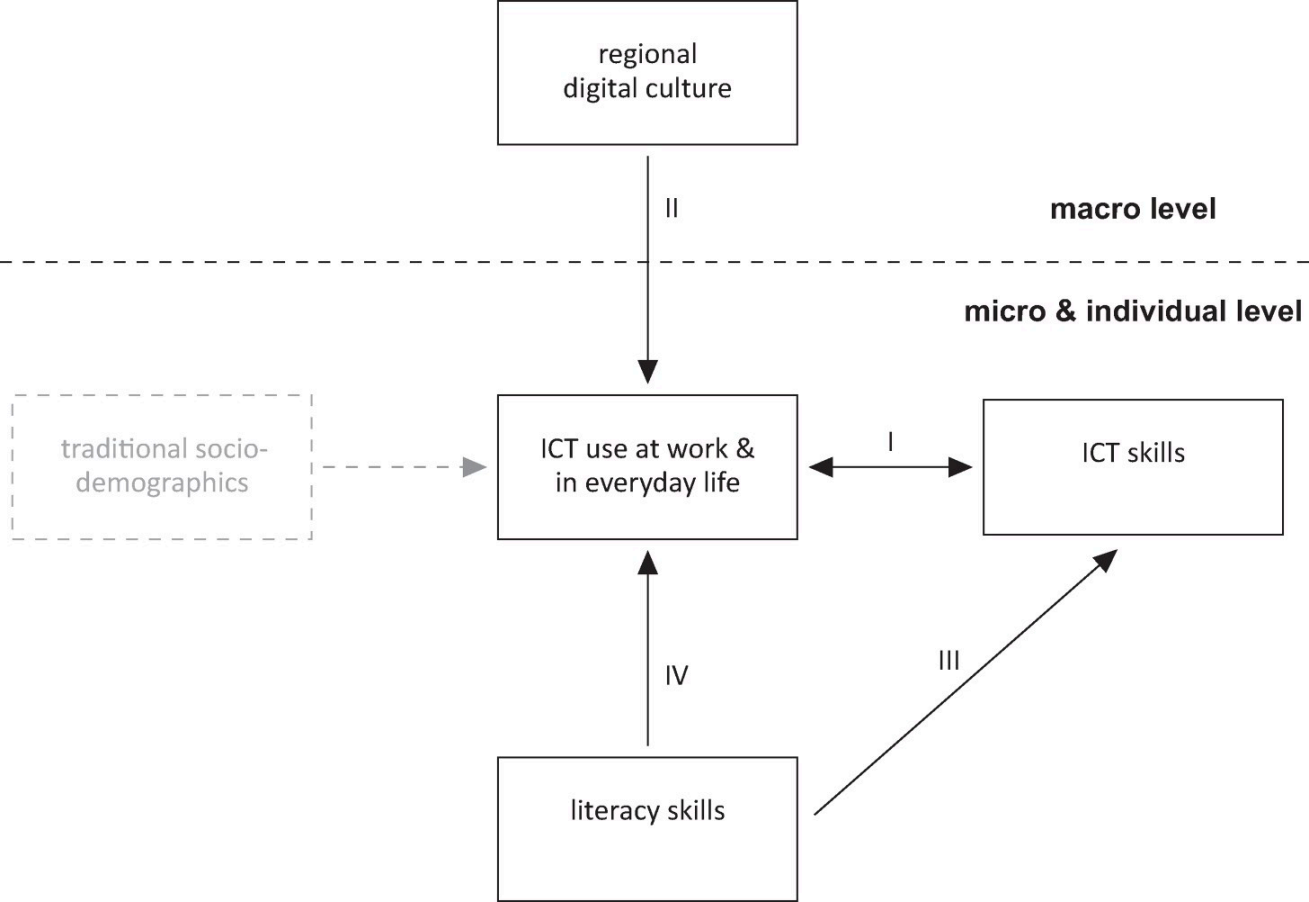
Conceptual framework identifying ICT use as a key prerequisite for the acquisition of ICT skills.

Our unified conceptual framework, shown in Fig 1, summarizes these key ideas. Consistent with practice engagement theory, our framework puts ICT use to the centre stage. To pay heed to the fact that ICT use does not operate in a vacuum but is multiply determined, our framework distinguishes between three levels relevant for adults’ ICT use and ICT skills: (1) the *individual* level (particularly represented by individuals’ literacy skills and educational level, but also other socio-demographic characteristics), (2) the level of *micro-contexts* (represented by the workplace and in everyday settings in which ICT use takes place), and the level of more distal *macro-contexts* (represented by digital culture at the regional level). The framework assumes that factors on the individual, micro-contextual and macro-contextual level influence the acquisition of ICT skills mainly through their influences on ICT use.

### An empirical application of the framework

In the following, we put our framework to an empirical test. For this purpose, we leverage the unique analytical potential of two representative German large-scale studies that offer objective measures of adults’ ICT skills, along with information on the other key components of our framework. Based on this framework, our core hypotheses are the following:

Hypothesis 1: ICT use on the job and/or in everyday life is positively interrelated with ICT skills (path I).
Hypothesis 2: Regional digital culture is positively related to individuals’ ICT skills by way of ICT use on the job and/or in everyday life (path I & path II).
Hypothesis 3: The positive association between literacy and ICT skills is in part mediated by individuals’ ICT use on the job and/or in everyday life (path IV & path I).
Hypothesis 4: Individuals with a high degree of literacy skills have higher ICT skills (path III).

## Method

### Data and samples

First, we draw on data of the first cycle of the “Programme for the International Assessment of Adult Competencies” (PIAAC, DOI 10.4232/1.12560, ZA5846, and DOI 10.4232/1.12560, ZA5846) in 2012. Second, we make use of the fifth wave in 2011/12 of the starting cohort 6 “Adults” of the “National Educational Panel Study” (NEPS, DOI 10.5157/NEPS:SC6:5.1.0). The data are public and available upon registration from the NEPS and PIAAC websites, respectively. Both data sets enable us to take into account the individual level, the level of micro-contexts (workplace and home) as well as the more distal macro level (regions). Especially the possibility of merging characteristics of the area of residence at the level of districts (“Landkreise,” NUTS-3) to the individual data makes these data unique with regard to our research question and enables us to investigate the interplay of regional conditions (digital culture), individual behaviour (ICT use) and ICT skills. However, concerning the occupational level, we are not able to distinguish between ICT use and relevant occupational conditions, as no occupational classification reflecting degrees of digitalization is available as yet. Therefore, our measure of using digital technologies on the job should be regarded as a conflated measure of individual action in context. In particular, the measure of ICT use on the job (described below) may often reflect characteristics of the job itself (in its layers of context) as well as characteristics of the individual in context.

#### PIAAC

PIAAC was initiated by the Organisation for Economic Co-operation and Development (OECD) and designed to provide internationally comparable measures of the cognitive abilities of the adult population aged between 16 and 65 years. While the whole study comprises data about 24 OECD and related countries, in our study, we only draw on the data for Germany. This restriction enables us to compare the results we obtain from PIAAC with those from NEPS.

The German sample of PIAAC comprises 5,465 persons. However, ICT skills are assessed only for persons reporting previous experience using computers, who consented to a computer-based skills assessment and who demonstrated basic capability using the computer keyboard and mouse). The missingness resulting from this routing process is informative in that it points to different stages of a “digital inclusion pathway” [28]. However, for this paper, we do not take such missing patterns into account, as no equivalent information is provided by NEPS. Hence, from the initial sample, we have valid information on the digital and literacy skills of 4,541 persons. Also, a large number of 1,578 cases must be excluded due to missing information about ICT use on the job, with 871 persons who currently were not employed. Further 90 persons provide no information on the use of ICT in everyday life, and 378 persons did not mention standard socio-demographic characteristics (gender, age, ethnicity, level of education). In the end, our analyses sample consists of 2,495 persons.

#### NEPS

The data from the starting cohort 6 of the NEPS were collected as part of the “Framework Programme for the Promotion of Empirical Educational Research” funded by the German Federal Ministry of Education and Research; as of 2014, the Leibniz Institute for Educational Trajectories at the University of Bamberg conducts the NEPS survey in cooperation with a nationwide network [67]. The current release of the study comprises a representative sample of 15,249 individuals aged 24 to 70 years of which 6,135 individuals had their ICT skills assessed in the fifth wave. From this sample, only 3,676 individuals also have information from literacy skills assessed in wave 3. The large number of missing values results from the randomized allocation of literacy and numeracy tests in wave 3 [68]. In addition, 2,786 cases also have valid information on ICT use on the job; no further missing values occur concerning ICT use in everyday life and the basic socio-demographic variables we use for control purposes.

### Individual and micro level measures

In what follows, we describe the relevant measures in PIAAC and NEPS we use to test the hypotheses derived from our theoretical framework empirically. Table 1 gives an overview of the basic descriptive statistic of these theoretically important variables and the socio-demographic characteristics used as controls.

**Table 1:** Basic statistics of individual-level variables.

	PIAAC		NEPS	
	mean/percent	S.D.	mean/percent	S.D.
*Sex*				
female	48.90		48.38	
male	51.10		51.62	
*Level of education*				
high (ISCED 5–6)	44.77		55.92	
medium (ISCED 3–4)	49.02		40.38	
low (ISCED 0–2)	6.21		3.7	
*Migration*				
German	83.85		84.82	
1st generation immigrant	9.10		5.78	
2nd generation immigrant	7.05		9.40	
Age	39.06	.24	49.19	9.45
ICT skills	293.27	1.07	.15	1.19
Literacy skills	288.08	.89	.24	1.28
*Using digital technologies in everyday life*				
zero to 20%	11.86		–	
more than 20% to 40%	15.79		–	
more than 40% to 60%	24.09		–	
more than 60% to 80%	27.41		–	
more than 80%	20.84		–	
several times a month or rarely	–		4.63	
several times a week	–		9.08	
daily or almost daily	–		86.29	
*Using digital technologies on the job*				
zero to 20%	22.77		–	
more than 20% to 40%	19.44		–	
more than 40% to 60%	23.77		–	
more than 60% to 80%	21.40		–	
more than 80%	12.63		–	
none	–		13.24	
one	–		34.60	
two	–		28.57	
three	–		11.56	
four	–		8.65	
five	–		3.37	
*N*	2,495		2,786	

#### ICT skills

In the assessment framework of PIAAC, ICT skills are conceptualized and measured by an individual’s proficiency in “problem-solving in technology-rich environments.” This PIAAC framework combines general skills in problem-solving and specific skills in using ICT. We label this combination of competencies “ICT skills.” The assessment of individuals’ ICT skills was computer-based, where the test situation corresponds to a real-life situation in which the computer is used to solve the problems raised by the test items. The items rely on everyday problems, which typically can be solved by using ICT (for example items, see OECD [69, pp. 53–55]).

Based on the data of all participating countries of PIAAC, the items were scaled using item response theory (IRT) producing a score ranging from zero to 500 points, with an average of 250 points and a standard deviation of 50 points. For each respondent ten plausible values were imputed, representing measurement error in the posterior distribution of their proficiency (for further information on IRT see [70]).

In line with PIAAC and other international large-scale assessments, NEPS defined ICT skills from a functional perspective that involves the knowledge and skills needed to manage everyday problems and to participate in society [71]. Using IRT, these assessments include Warm’s mean weighted likelihood estimations (WLEs) of individuals’ ICT skills as point estimates of individuals given their item responses [72]. Despite the similarities of NEPS and PIAAC on a theoretical level, their assessments of ICT skills are quite different. One major difference concerns the test mode: While the test in PIAAC was a computer-based assessment, the NEPS test was paper-based with simple multiple choice and true-false items. This comes along with differences in how they covered the variety of item difficulty levels. All items in NEPS require only one step, and no conclusions are necessary to answer the questions. In contrast to PIAAC, where ICT skills were assessed as individuals’ proficiency in problem-solving in the context of ICT use, the NEPS tests pure technological as well as information skills (for sample items, see [73]).

#### ICT use

Both PIAAC and NEPS include extensive information on self-reported ICT use on the job and in everyday life. Both measures depict individuals’ practice engagement within predefined micro-contexts. Within PIAAC, the ICT use on the job variable is only available for respondents who were employed at the time of the interview or during the year preceding the interview; in NEPS it is only available for currently employed individuals.

In order to measure ICT use on the job and in everyday life, PIAAC respondents were asked how often they use different kinds of applications. The scale ranges from 1 (“never”) to 5 (“every day”). Based on the single items on ICT use on the job and in everyday life, PIAAC provides WLEs, which are normalized relative to the most active user in the dataset. We use the discrete form of this index in our analyses, which consist of five percentile groups: “zero to 20%,” “more than 20% to 40%,” “more than 40% to 60%,” “more than 60% to 80%,” “more than 80%.”

For the NEPS data, we constructed a sum score for ICT use on the job based on six items answered with yes or no; we calculated the score only for those who answered at least four out of the six questions. This item battery was surveyed in wave 4. The data on individuals’ self-reported use of ICT in everyday life were compiled with the question of how often the respondent has used the computer in the last year: “daily or almost daily,” “several times a week,” “several times a months,” “rarely,” or “never.” We combined the last two groups due to a limited number of cases within each category.

#### Literacy skills

In order to measure the prerequisites of ICT use and digital skill acquisition at the individual level, we draw on individuals’ literacy skills that are believed to be fundamental to both ICT use and ICT skills. In PIAAC, literacy skills were always assessed in a computer-based mode for individuals whose ICT skills were assessed. Individual proficiency measures (as plausible values) were derived in the same way with IRT as done for ICT skills. In the NEPS study, literacy skills were tested in a paper-based test mode in wave 3, two waves before the assessment of their ICT skills. The items were scaled the same way as for ICT skills, using IRT.

### Regional digital culture

**Fig 2:**
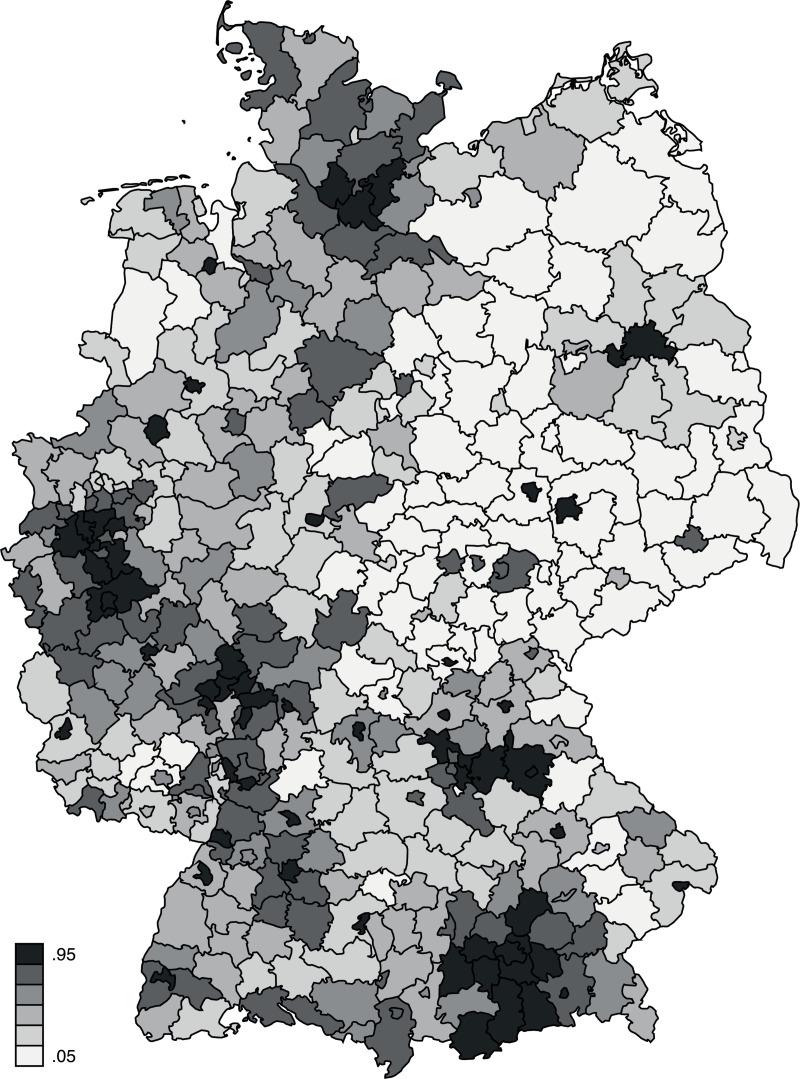
Regional distribution of digital culture,.de-domains per capita 2012 [74]. (A) © GeoBasis-DE/BKG (2011).

In order to capture the extent to which the regional context harbors a “digital culture” that demands and encourages ICT use, we used data on the number of in Germany registered internet domain,.de-domains, in the year 2012 at the level of German districts (Landkreise, NUTS-3), their distribution is shown by Fig 2. “.de” is the country code top-level domain for the Federal Republic of Germany. The.de-domains are administered by the main domain registry DENIC eG, which is a German non-profit cooperative. The data are part of the publicly available regional database Germany published by the Federal Statistical Office and the Statistical Offices of the Länder [74]. This regional indicator was a suitable proxy for the “digital culture” of a region because it captures ICT-related practices—namely, registering internet domains as a prerequisite for producing and publishing content online—at the regional level. It also reflects the unequal distribution of access to the internet and the economic makeup of the region to the extent that these factors relate to social practices in terms of content production at the regional level [56, 57].

### Analyses

In order to investigate the association of regional digital culture as well as individual resources and ICT skills, we draw on generalized linear regression models. We follow a stepwise approach to analyse the mediating role of ICT use. As the data are structured hierarchically, we conduct both kinds of analyses with a Huber-White sandwich estimator of variances in order to obtain cluster-robust standard errors [75]. PIAAC additionally requires taking the IRT approach into account, as for each person ten plausible values are available which represent their proficiency distribution. For this purpose, we treat the plausible values for ICT skills as multiply imputed values.

When analysing the relevance of using digital technologies for adults’ ICT skills, we are faced with the problem of endogeneity resulting from reverse causality, i.e., individuals who use digital technologies more often show higher values in ICT skills and vice versa. This reciprocal relationship is also predicted by the practice engagement theory [5]. With cross-sectional data, we are not able to make causal inferences.

## Results

**Fig 3:**
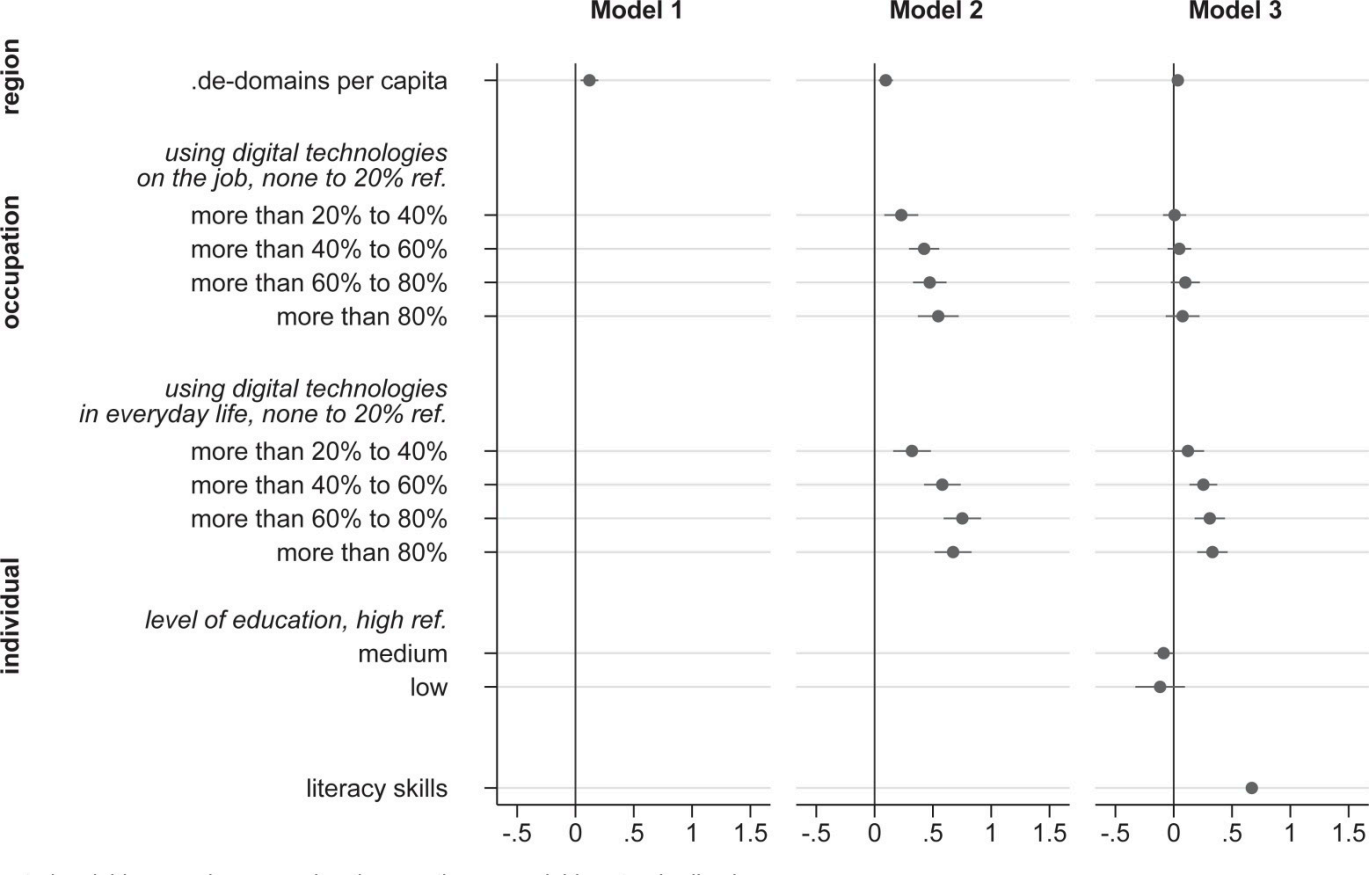
ICT skills regressed on individual and contextual factors (PIAAC). (A) Control variables: gender, age, migration. (B) Continuous variables standardized, cluster robust standard errors, 95% confidence intervals. (C) PIAAC Germany 2012, N(individuals) 2,495, N(regions) 245.

**Fig 4:**
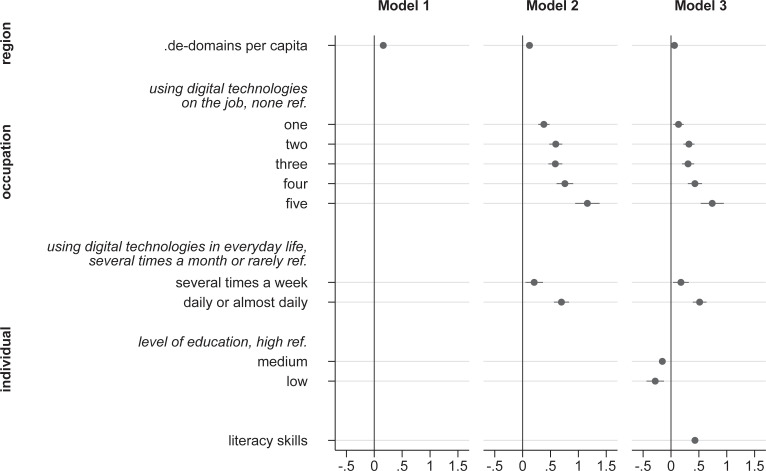
ICT skills regressed on individual and contextual factors (NEPS). (A) Control variables: gender, age, migration. (B) Continuous variables standardized, cluster robust standard errors, 95% confidence intervals. (C) NEPS starting cohort 6 2012/13, N(individuals) 2,786, N(regions) 259.

We present the results of our analyses separately for PIAAC in Fig 3 and NEPS in Fig 4. We control for socio-demographic characteristics, including gender, age, and migration. The regression results for these control variables are omitted from the figures for simplicity; the complete results of the full Models are in S1 Table. Because all continuous predictor variables are standardized, including the dependent variable, the regression coefficients in Figs 3 and 4 should be interpreted as changes per standard deviation.

All in all, the results from both data sources, PIAAC, and NEPS, give a similar picture: Starting with investigating the association between internet domain registrations per capita at the regional level and individuals’ ICT skills (Model 1 of Figs 3 and 4), the results indicate only a moderate, albeit not negligible positive relationship. In the next step, we introduce individuals’ ICT use on the job and in everyday life. In line with Hypothesis 1, ICT use in both contexts is strongly related to individuals’ ICT skills (Model 2 of Figs 3 and 4). However, introducing these variables does not lead to a substantial reduction of the regression weight for.de-domains per capita. Therefore, in contrast to Hypothesis 2, the use of digital technologies within both micro-contexts, workplace and everyday life, seems to play a less significant role for the association between regional digital culture and individuals’ ICT skills (the regression results for the sources of ICT use at workplace can be found in the appendix in S2 Table and for ICT use in everyday life in S3 Table.

The sources of the association between ICT use and ICT skills can be revealed after taking into account individuals’ literacy skills (Model 3 of Figs 3 and 4). Also, we introduced individuals’ level of formal education which tends to show no incremental association with ICT skills. Considering individuals’ literacy skills and level of formal education leads to a decline of ½ of the initially large regression coefficients for ICT use on the job and in everyday life. These results support Hypothesis 3, which stated that the positive association between literacy and ICT skills is in part mediated by individuals’ ICT use on the job and/or in everyday life. However, ICT use on the job and in everyday life is still incrementally related to ICT skills after controlling for education and literacy skills. Only in PIAAC, the regression weights for ICT use on the job are no longer statistically significant.

Literacy skills are not only indirectly correlated with ICT skills through ICT use; they are incrementally associated with individuals’ ICT skills (hypothesis 4). In the PIAAC data, for instance, a one standard deviation change of literacy skills is associated with a .67 standard deviations increase in ICT skills. Introducing literacy skills further reduces the regression weight for regional digital culture. As the regression coefficients for the educational level show, medium and low levels of education are associated with successively lower ICT skills.

## Discussion

The rapid digital transformation that is taking place across the globe has led to a surge in the importance of ICT skills for individuals and societies alike. In this context, a question of high relevance to researchers, policymakers, and practitioners is why some adults are better able to successfully deal with modern ICT than others. In the present study, building on previous research and theorizing from different disciplines, we developed a new guiding framework that aims to explain individual differences in ICT skills. Borrowing from practice engagement theory [5] and related theoretical ideas, our framework highlights social practices in terms of ICT use as key prerequisites to the acquisition of ICT skills. Building on the insight that most adults in current cohorts received little formal training regarding ICT, we argued that adults mainly acquire ICT skills through informal learning processes, that is, through the continued application of ICT at the workplace or in everyday life. We conceive such ICT-related social practices to be embedded in multi-layered micro- and macro-contexts on the one hand and contingent on individuals’ literacy skills on the other hand. Thus, contextual opportunity structures and individuals’ literacy skills jointly shape the acquisition of ICT skills mainly through their influence on the frequency and intensity of ICT use.

We empirically tested this framework with two recent German large-scale surveys, NEPS and PIAAC. These surveys offered unique analytic potential for this endeavour, as both offer objective—and comparable—measures of adults’ ICT skills in large and diverse samples of adults. These analyses yielded three main findings. First, in line with our framework’s main tenet, ICT use at the workplace and in everyday life emerged as strong correlates (i.e., potential determinants) of ICT skills in both surveys. This suggests that individuals’ ICT skills are inextricably interwoven with the learning opportunities afforded by the micro-contexts of work and everyday life.

Second, adults’ literacy skills are another potent correlate of ICT skills in both data sets, in addition to the already well established sources of individual differences in ICT skills (education, age, sex, and migration). When literacy skills were added to the equation, the coefficients for ICT use dropped considerably, in line with the idea literacy skills have both direct associations with ICT skills and indirect associations through ICT use. This pattern of findings is consistent with our assumption that literacy skills are indispensable for successfully dealing with ICT and the acquisition of ICT skills.

Third, we also found some (albeit more limited) support for the idea that social practices in the macro-context are related to individuals’ ICT skills, again mainly through individuals’ ICT use at work and in everyday life. More specifically, living in a region with a stronger digital culture—as proxied by the internet domain registration rate, one aspect of digital culture in the sense of content production—is associated with higher ICT skills. The shrinkage of the coefficient when ICT use is added to the model indicates this may be partly due to adults in high registration regions being more likely to use ICT.

Taken together, our findings lend support to the idea borrowed from practice engagement theory that ICT use at work and in everyday life is of paramount importance for the acquisition of ICT skills. At the same time, the findings direct attention to literacy skills as a prerequisite to both ICT use and ICT skills. Without a sufficient level of literacy, adults will be unlikely to fully meet the challenges in acquiring the ICT skills necessary to successfully deal with modern ICT at work and beyond.

### Limitations and directions for future research

Our empirical analyses are certainly not without limitations. First, both data sources we used were cross-sectional. The reported associations can, therefore, not be interpreted as causal, even though some directions of influence are certainly more likely than others (e.g., ICT skills cannot influence age or gender). Especially with regard to the relationship between ICT use and ICT skills, we face the potential problem of reverse causality. Tackling this issue is only possible with longitudinal data and/or an instrumental variable approach, which both would greatly enhance the possibility for causal inferences. However, as expressed by the two-sided arrow in Fig 1, we deem it very likely that the relationship between ICT use and ICT skills is reciprocal. That is, individuals will acquire ICT skills through ICT use—and those who possess higher ICT skills will use ICT more frequently. Nonetheless, future studies that track the development of ICT skills over time—ideally from childhood into adulthood—are needed to better understand not only the causal associations but also the temporal dynamics of the relationships stipulated by our framework.

Second, we emphasize that we were not able to disentangle structural opportunities from individuals’ ICT use, as this would require further measures related to the micro-contexts workplace and everyday life settings, such as partners’ ICT use and skills or the degree of digitalization of occupation. Regrettably, the NEPS and PIAAC data contain no further information on partners and, as yet, no occupational classification relevant to digitalization is available.

Third, although literacy skills are a strong predictor of adults’ digital skills, they cannot perfectly explain them. This speaks to the fact that ICT skills are a multifaceted construct that cuts across other skill domains. Almost anything can be done with digital technologies (even in early childhood where literacy skills are absent or less developed): Play activities, learning activities, creative activities, or social activities, and these activities require different further skills (e.g., mathematical skills). Further research is needed that focuses on the factors that are relevant for using digital technologies in a way that is detached from written language.

Fourth, the moderate observed relationship between individuals’ skills and regional digital culture (through ICT use) might result from the specific aggregate level used in our analyses. Previous research has shown that the results of regional data analyses are highly sensitive to the scale and zoning of the chosen regionalization; this phenomenon is known as the modifiable areal unit problem [MAUP; 76]. However, data on.de-domains per capita that we used to proxy digital culture are only available for German districts (Landkreise, NUTS-3) or more highly aggregated regions. Although districts are small-scale regions, they might be too large to operationalize individuals’ actual horizon for action. In addition, while.de registrations have been shown to be a good proxy for social practices in terms of content production, they may not entirely represent what constitutes digital culture (e.g., corporate culture, information culture, communication culture), and therefore, correlations between regional influences and ICT skills may be underestimated. Further studies are needed to compare correlations between individuals’ skills and regional digital culture at different regional levels and using different measures for regional digital culture. These findings can be a valuable starting point for future research.

### Conclusion

Our study contributes to the literature on the origins of individual difference in adults’ ICT skills in several ways. In line with our framework, our findings highlight that ICT skills do not emerge in a vacuum but are strongly related to individuals’ ICT use. That is, adults’ ICT skills are largely associated with “learning by doing” at home and work. At the same time, our framework and findings direct attention to the individual and to a lesser degree contextual preconditions of ICT use. Above and beyond well-established socio-demographic characteristics, our findings identify literacy skills as a key precondition for both ICT use and ICT skills. Literacy skills show both direct associations with ICT skills and indirect associations through ICT use at the workplace and in everyday life. Moreover, our findings suggest that regional macro-context co-shape ICT skills, mainly through their influence on ICT use in the micro-contexts of home and workplace. However, micro-level factors, such as literacy skills and ICT use, were found to be more strongly related to ICT skills than regional macro-level factors. This suggests that future research and policies aimed at narrowing the “digital divide” should pay particularly attention to the fundamental role of individual literacy skills in shaping patterns of ICT use, which in turn might be the key vehicle of digital skill acquisition. Although macro-level factors appeared to be less important for ICT skills, our study points to the importance of a stimulating environment for ICT use and ICT skills, which may be less important at the regional level than at the lower level of companies or communities. This should also be the subject of future research in order to establish viable targets for policies and interventions aimed at fostering ICT skills and decreasing social inequality therein.

## Supporting information

S1 Table ICT skills regressed on individual and contextual factors, full regression results for PIAAC and NEPS. (DOCX)

S2 Table ICT use on the job regressed on individual and contextual factors, ordered logistic regression (logit coefficients), PIAAC and NEPS. (DOCX)

S3 Table ICT use in everyday life regressed on individual and contextual factors, ordered logistic regression (logit coefficients), PIAAC and NEPS. (DOCX)
