# Supplementary material for: Sources of individual differences in adults’ ICT skills: A large-scale empirical test of a new guiding framework
Source: PLoS One. 2021 Apr 19;16(4):e0249574. doi: 10.1371/journal.pone.0249574 (PMC8054998; doi:10.1371/journal.pone.0249574)
Supplement: S1 Table — (DOCX) [file pone.0249574.s001.docx]

**S1 Table. ICT skills regressed on individual and contextual factors, full regression results for PIAAC and NEPS.**

|  | PIAAC |  |  | NEPS |  |
| --- | --- | --- | --- | --- | --- |
|  | β | S.E. |  | β | S.E. |
| *Sex, male ref.* | –.041 | .033 |  | –.272 | .027 |
| *Education level, high (ISCED 5–6) ref.* |  |  |  |  |  |
| Medium (ISCED 3–4) | –.086 | .039 |  | –.154 | .030 |
| Low (ISCED 0–2) | –.116 | .102 |  | –.282 | .079 |
| *Migration, German ref.* |  |  |  |  |  |
| 1st generation immigrant | –.206 | .077 |  | –.125 | .048 |
| 2nd generation immigrant | .008 | .064 |  | .005 | .048 |
| Age | –.174 | .022 |  | –.270 | .014 |
| Literacy skills | .669 | .020 |  | .429 | .015 |
| *ICT use in everyday life, 0 to 20 % ref.* |  |  |  |  |  |
| More than 20 % to 40 % | .121 | .068 |  | – |  |
| More than 40 % to 60 % | .254 | .059 |  | – |  |
| More than 60 % to 80 % | .309 | .064 |  | – |  |
| More than 80 % | .332 | .064 |  | – |  |
| *ICT use in everyday life,  several times a months or rarely ref.* |  |  |  |  |  |
| Several times a week | – |  |  | .178 | .071 |
| Daily or almost daily | – |  |  | .513 | .061 |
| *ICT use on the job, zero to 20 % ref.* |  |  |  |  |  |
| More than 20 % to 40 % | .007 | .049 |  | – |  |
| More than 40 % to 60 % | .047 | .050 |  | – |  |
| More than 60 % to 80 % | .100 | .060 |  | – |  |
| More than 80 % | .076 | .070 |  | – |  |
| *ICT use on the job, none ref.* |  |  |  |  |  |
| One | – |  |  | .135 | .047 |
| Two | – |  |  | .321 | .050 |
| Three | – |  |  | .306 | .055 |
| Four | – |  |  | .429 | .064 |
| Five | – |  |  | .739 | .105 |
| .de-domains per capita | .035 | .016 |  | .062 | .016 |
| Constant | –.188 | .069 |  | –.484 | .065 |
| Notes: continuous variables standardized, cluster robust standard errors, 95% confidence intervals, PIAAC Germany 2012, N(individuals) 2,495, N(regions) 245, NEPS starting cohort 6 2012/13, N(individuals) 2,786, N(regions) 259 | | | | | |
