# Supplementary material for: Sources of individual differences in adults’ ICT skills: A large-scale empirical test of a new guiding framework
Source: PLoS One. 2021 Apr 19;16(4):e0249574. doi: 10.1371/journal.pone.0249574 (PMC8054998; doi:10.1371/journal.pone.0249574)
Supplement: S2 Table — (DOCX) [file pone.0249574.s002.docx]

**S2 Table. ICT use on the job regressed on individual and contextual factors, ordered logistic regression (logit coefficients), PIAAC and NEPS.**

|  | PIAAC |  |  | NEPS |  |
| --- | --- | --- | --- | --- | --- |
|  | β | S.E. |  | β | S.E. |
| Sex, male ref. | –.18 | (.07) |  | –.64 | (.07) |
| Education level, high (ISCED 5–6) ref. |  |  |  |  |  |
| Medium (ISCED 3–4) | –.61 | (.08) |  | –.70 | (.09) |
| Low (ISCED 0–2) | –1.33 | (.23) |  | –.78 | (.22) |
| Migration, German ref. |  |  |  |  |  |
| 1st Generation immigrants | –.52 | (.16) |  | –.49 | (.14) |
| 2nd Generation immigrants | .11 | (.17) |  | –.12 | (.10) |
| Age | .10 | (.04) |  | –.03 | (.03) |
| Literacy skills | .42 | (.05) |  | .26 | (.04) |
| .de-domains per capita | .05 | (.05) |  | .07 | (.03) |
| Cut1 | –1.84 | (.08) |  | –2.66 | (.08) |
| Cut2 | –.80 | (.07) |  | –.74 | (.07) |
| Cut3 | .21 | (.07) |  | .60 | (.07) |
| Cut4 | 1.58 | (.08) |  | 1.46 | (.08) |
| Cut5 | – |  |  | 2.86 | (.12) |
| N(individuals) | 2,495 |  |  | 2,786 |  |
| N(regions) | 245 |  |  | 259 |  |
| Notes: continuous variables standardized; cluster robust standard errors, 95% confidence intervals. | | | | | |
