# Supplementary material for: Sources of individual differences in adults’ ICT skills: A large-scale empirical test of a new guiding framework
Source: PLoS One. 2021 Apr 19;16(4):e0249574. doi: 10.1371/journal.pone.0249574 (PMC8054998; doi:10.1371/journal.pone.0249574)
Supplement: S3 Table — (DOCX) [file pone.0249574.s003.docx]

**S3 Table. ICT use in everyday life regressed on individual and contextual factors, ordered logistic regression (logit coefficients), PIAAC and NEPS.**

|  | PIAAC |  |  | NEPS |  |
| --- | --- | --- | --- | --- | --- |
|  | β | S.E. |  | β | S.E. |
| Sex, male ref. | –.44 | (.07) |  | –.65 | (.10) |
| Education level, high (ISCED 5–6) ref. |  |  |  |  |  |
| Medium (ISCED 3–4) | –.39 | (.08) |  | –.59 | (.12) |
| Low (ISCED 0–2) | –.37 | (.21) |  | –.78 | (.27) |
| Migration, German ref. |  |  |  |  |  |
| 1st Generation immigrants | –.22 | (.17) |  | –.14 | (.23) |
| 2nd Generation immigrants | –.20 | (.14) |  | .03 | (.21) |
| Age | –.25 | (.04) |  | –.25 | (.05) |
| Literacy skills | .38 | (.05) |  | .62 | (.07) |
| .de-domains per capita | .02 | (.06) |  | .27 | (.06) |
| Cut1 | –2.53 | (.09) |  | –3.99 | (.13) |
| Cut2 | –1.45 | (.09) |  | –2.72 | (.11) |
| Cut3 | –.31 | (.09) |  | – |  |
| Cut4 | .98 | (.09) |  | – |  |
| N(individuals) | 2,495 |  |  | 2,786 |  |
| N(regions) | 245 |  |  | 259 |  |
| Notes: continuous variables standardized; cluster robust standard errors, 95% confidence intervals. | | | | | |
